# Feasibility and preliminary efficacy of an infant preemptive intervention for prodromes of autism spectrum disorder (ASD) delivered within an Italian tertiary hospital

**DOI:** 10.3389/frcha.2026.1776508

**Published:** 2026-06-02

**Authors:** Costanza Colombi, Natasha Chericoni, Eugenia Conti, Ilaria Colombino, Giulia Guainai, Benedetta Riva, Fabio Apicella, Sara Calderoni, Andrea Guzzetta

**Affiliations:** 1 IRCCS Stella Maris Foundation, Pisa, Italy; 2 Department of Clinical and Experimental Medicine, University of Pisa, Pisa, Italy

**Keywords:** autism spectrum disorder, feasibility, infants, preemptive intervention, prodromal signs, telehealth

## Abstract

**Background:**

Autism spectrum disorder (ASD) is a neurodevelopmental condition with early-emerging social-communication atypicalities, yet diagnosis and access to specialized intervention often occur years after prodromal signs first appear. Preemptive, parent-mediated Naturalistic Developmental Behavioral Interventions (NDBIs) delivered in the first year of life have shown promise, but evidence for their feasibility and clinical impact within routine public services remains limited. This pilot study examined the feasibility, acceptability, and preliminary efficacy of FIRRST (Fostering Infant Responsivity and Reciprocity—Support to Thrive), a telehealth NDBI program for infants under 15 months with early signs of ASD, implemented in an Italian tertiary child neuropsychiatric hospital.

**Methods:**

Infants younger than 15 months were recruited following identification as at elevated likelihood for ASD on the Social Attention and Communication Surveillance–Revised (SACS-R) and exclusion of neurological conditions determined by specialists. Eligible families (*N* = 30) participated in weekly, 1-h, telehealth, parent-mediated FIRRST sessions for six months, delivered by trained clinicians and supporting social engagement, communication, play, motor development, and environmental enrichment within everyday routines. Feasibility and acceptability outcomes included recruitment, retention, and a parent satisfaction questionnaire, while caregiver implementation of NDBI strategies (MONSI-CC) was the primary clinical outcome; secondary outcomes were children's ASD symptoms (BOSCC), developmental level (Griffiths III), adaptive functioning (Vineland-II), and parental stress (PSI-4), assessed at baseline (T0), 3 months (T1), and post-intervention (T2) using repeated-measures ANOVA.

**Results:**

Of 44 screened families, 30 were enrolled and 28 completed the full program, yielding an attrition rate of 6.7%; dropouts were attributed to practical constraints rather than intervention-related factors. Parental satisfaction ratings were predominantly positive across domains of perceived usefulness, therapeutic alliance, and impact on the child's enjoyment and social engagement. Caregivers showed significant, progressive improvements from T0 to T2 across all MONSI-CC domains and total score, paralleled by significant reductions in BOSCC social-communication and restricted and repetitive behavior scores and gains in Griffiths III and Vineland-II domain and composite scores, whereas PSI-4 total and subscale scores did not show significant change over time. Among the 17 children eligible for the ADOS-2 Toddler Module at T2, mean total and domain scores were in the low range, consistent with relatively mild ASD symptom expression at post-intervention.

**Discussion:**

FIRRST was feasible and acceptable for families of infants with ASD prodromes within a public child neuropsychiatric hospital, with high retention and strong caregiver endorsement of the telehealth, parent-mediated format. The pattern of increased caregiver NDBI strategy fidelity alongside reductions in early ASD symptoms and improvements in developmental and adaptive functioning suggests that a very-early, family-centered, telehealth NDBI may positively support early trajectories for a subset of infants at elevated likelihood for ASD. These pilot findings support the implementation of a larger multicenter randomized controlled trial rigorously evaluating FIRRST and underscore the potential of first-year preemptive intervention embedded in public health systems.

## 1 Introduction

Autism spectrum disorder (ASD) is a heterogeneous neurodevelopmental condition characterized by early-emerging atypicalities in social communication and restricted and repetitive behaviors, with lifelong impact on individuals, families, and healthcare systems (1, 2). Although converging evidence indicates that behavioral and neurophysiological markers of ASD can be detected within the first year of life, the mean age of diagnosis in routine clinical practice remains close to 4 years, resulting in a substantial delay between the emergence of prodromal signs and access to specialized intervention (2–4). This latency is particularly critical given that interventions initiated before the age of 2 years are associated with more favorable trajectories in language, social communication, adaptive functioning, and ASD symptom severity than the same programs begun later in development (5, 6).

Over the past decade, longitudinal and prospective studies have clarified that early-emerging signs of ASD can be observed in the first months of life, well before a formal diagnosis is typically made. Subtle but reliable indicators include diminished or declining eye contact, reduced social smiling and reciprocity, atypical response to name, limited use of gestures, and unusual patterns of object exploration and motor development (4, 7, 8). Children who later receive an ASD diagnosis often show a divergence from typical trajectories in social attention and interactive behaviors between 2 and 12 months, while caregivers frequently report concerns about passivity, lack of engagement, and repetitive motor behaviors during the first year of life (4, 6, 9). Tools such as the Social Attention and Communication Surveillance–Revised (SACS-R) have demonstrated good accuracy in detecting these early social-communication atypicalities in community and clinical settings, thereby offering a pragmatic pathway to identify infants with ASD prodromes who may benefit from preemptive intervention (10, 11).

In this context, preemptive interventions targeting infants who already show early social-communication atypicalities have gained increasing empirical support. Several studies including randomized trials of parent-mediated models such as the Infant Start and the iBASIS-VIPP have demonstrated that working with caregivers during the first year of life can reduce ASD symptom severity, enhance developmental outcomes, and lower the likelihood of an ASD diagnosis at preschool age, suggesting that disability associated with the full-blown syndrome may be attenuated for a proportion of children (12, 13). However, most existing preemptive programs have been tested in highly specialized research settings, and few data are available on their feasibility, acceptability, and initial efficacy within routine services (9, 13).

Naturalistic Developmental Behavioral Interventions (NDBIs) have emerged as a leading framework for early ASD intervention, integrating developmental principles with strategies from applied behavior analysis and emphasizing play-based, child-led learning embedded in everyday interactions (14). Within the Italian public health system, several studies have documented the feasibility and positive impact of NDBI models derived from the Early Start Denver Model (ESDM), both in therapist-delivered formats and in parent-mediated versions such as Parent-ESDM, which enhance caregivers’ use of NDBI strategies and are associated with improvements in children's core ASD behaviors (15–17). In parallel, an Italian case report of a symptomatic infant who received an Infant Start–based preemptive intervention from 6 months of age highlighted the clinical plausibility of acting within the first year of life, with progressive normalization of developmental scores and absence of ASD diagnosis at 32 months (9).

Building on this body of work, FIRRST (Fostering Infant Responsivity and Reciprocity—Support to Thrive) was developed as a family-centered, telehealth-delivered NDBI program specifically designed for infants under 15 months who show ASD prodromes (9, 13, 18). FIRRST extends prior Italian experience with Parent-ESDM and Infant Start by combining targeted coaching on social-communication and play with developmental motor support and environmental enrichment, and by embedding intervention within family routines and cultural practices (9, 12, 14, 17). Before implementing a large multicenter randomized controlled trial comparing FIRRST with a Parent Education control condition (ClinicalTrials.gov Identifier: NCT06817746), it was essential to establish the feasibility, acceptability, and initial clinical impact of this model within routine services.

The present study reports on a 6-month telehealth FIRRST pilot conducted at an Italian tertiary neuropsychiatric hospital, with infants identified at high likelihood for ASD based on the Social Attention and Communication Surveillance–Revised (10, 11). The primary objective was to evaluate feasibility and acceptability, indexed by recruitment, retention, parental satisfaction, and changes over time in caregivers' implementation of NDBI strategies. Secondary objectives were to examine, children's changes over time in ASD core symptoms, developmental functioning, adaptive behavior, and parental stress, thereby providing preliminary data to inform the design and expected effect sizes of the ongoing FIRRST randomized trial.

## 2 Materials and methods

### 2.1 Procedures

The study was carried out at the IRCCS Fondazione Stella Maris (FSM), Pisa, Italy, in accordance with the standards for good ethical practice of the Declaration of Helsinki, and was approved by the Pediatric Ethics Committee of the Meyer Hospital on 21 February 2023 (protocol number: VIITA23 No. 1, dated 17.01.2023). Written informed consent was obtained from all the children's parents. Families enrolled in the project participated in a 6-month telehealth parent-mediated intervention. Data on parents' use of NDBI strategies and on children's developmental outcomes were collected 1 week prior to the beginning of the intervention (T0), after 3 months from the beginning of the intervention (T1), and at the end of the intervention, in the two weeks following the last treatment session (T2).

### 2.2 Participants

Participants were assessed by a multidisciplinary team of autism specialists and enrolled in the study based on the following criteria: 1. Children under 15 months of age; 2. ASD likelihood-range score on the Social Attention and Communication Surveillance–Revised (SACS-R); 3. Agreement to participate in the intervention once a week over a period of 6 months; 4. Italian is one of the languages spoken at home. Exclusion criteria included: 1. Known genetic disorders; 2. Known perinatal brain damage or neurological conditions (e.g., seizures); 3. Significant chronic medical conditions; 4. Severe visual, hearing and/or motor impairments.

**Figure 1:**
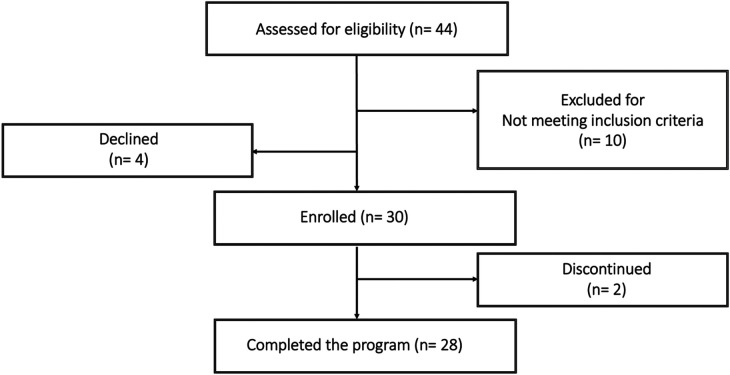
Flowchart of participant enrollment.

Forty-four families contacted our research team and were screened for eligibility. Ten children were excluded for not meeting the inclusion/exclusion criteria, and two eligible families declined participation, resulting in a final sample of 30 participants, of whom 28 completed the program (see Figure 1 for the participant enrollment flowchart).

At baseline, the thirty infants enrolled in this study (M/F = 20/10; age: M [SD] = 8.05 [2.82] range 3–13 months) were administered a standardized protocol to evaluate autism prodromes (SACS-R), child development (Griffiths III), adaptive behavior (Vineland-II) (See Table 1 for an overview of participants' demographic and clinical characterization).

**Table 1:** Clinical and demographic characteristics of enrolled families.

Infants’ characterization	*n*. 30	Parents’ characterization	Mothers	Fathers
M/F	20/10	Age M (SD)	35.88 (3.82)	38.30 (5.31)
Age in months, M (SD)	8.05 (2.82)	Range (years)	28–45	31–51
Clinical Assessment	M (SD)	Educational level		
SACS-R		Doctoral degree	3.33% (*n* = 1)	3.33% (*n* = 1)
Key Items	4.46 (.78)	University degree	73.33% (*n* = 22)	40.00% (*n* = 12)
Griffiths III		High school diploma	23.33% (*n* = 7)	40.00% (*n* = 12)
Foundations of Learning	96.48 (13.46)	Middle School diploma	.00% (*n* = 0)	16.67% (*n* = 5)
Language and Communication	83.79 (12.68)	Ethnicity		
Eye and Hand Coordination	92.69 (10.49)	Italian	96.67% (*n* = 29)	96.67% (*n* = 29)
Personal-Social-Emotional	84.31 (11.32)	Not italian	3.33% (*n* = 1)	3.33% (*n* = 1)
Gross Motor	98.28 (11.12)	Race		
General Development	89.38 (9.57)	White	100% (*n* = 30)	.00% (*n* = 0)
Vineland-II		American Indian or Alaska Native	.00% (*n* = 0)	.00% (*n* = 0)
Communication	84.10 (7.27)	Asian	.00% (*n* = 0)	.00% (*n* = 0)
Daily Living Skills	88.93 (10.91)	Black or African American	.00% (*n* = 0)	.00% (*n* = 0)
Socialization	86.47 (10.67)	Hispanic or Latino	.00% (*n* = 0)	3.33% (*n* = 1)
Motor Skills	92.03 (12.76)	Hawaiian or Other Pacific Islander	.00% (*n* = 0)	.00% (*n* = 0)
Adaptive Behavior Composite	87.77 (9.72)	More than one Race	.00% (*n* = 0)	.00% (*n* = 0)
Distribution of ASD risk factors		Geographic origin		
1. Pregnancy/Birth Complications	30.0% (*n* = 9)	North-est Italy	20.00% (*n* = 6)	
2. Medically Assisted Procreation	30.0% (*n* = 9)	North-west Italy	26.67% (*n* = 8)	
3. ASD Sibling in the family	20.0% (*n* = 6)	Central Italy	33.33% (*n* = 10)	
4. Prematurity (<37 weeks)	10.0% (*n* = 3)	South Italy and Islands	20.00% (*n* = 6)	
5. Small for Gestational Age (<10°)	3.3% (*n* = 1)			
At least 1/5 risk factors	66.7% (*n* = 20)			
Other Medical/Health issues				
Plagiocephaly/Dyscrania	50.0% (*n* = 15)			
Motor Asymmetries	96.7% (*n* = 29)			

At enrollment, all infants had scores in the ASD likelihood range on the SACS-R and presented with early social-communication atypicalities, such as reduced eye contact, limited social smiling and reciprocity, inconsistent response to name, reduced use of gestures and socially directed vocalizations, and atypical object exploration or motor patterns, consistent with prodromal signs of ASD described in the literature. During the intake interview, parents typically reported seeking specialist advice because they had noticed subtle but persistent differences in their infant's social engagement and communication during everyday interactions, sometimes accompanied by broader developmental or behavioral concerns. Families accessed our tertiary neuropsychiatric hospital either via self-referral, following parental recognition of these early signs (70%), or following referral from their family pediatrician or other health professionals who had identified similar concerns (30%).

### 2.3 Intervention

FIRRST (Fostering Infant Responsivity and Reciprocity—Support to Thrive) is a family-centered, telehealth-delivered program based on Naturalistic Developmental Behavioral Interventions (NDBIs), based on principles of Infant Start (12) and iBASIS (13), combined with developmental motor therapy and environmental enrichment. It targets early ASD prodromes such as reduced eye contact, communicative intent, social orientation, atypical object exploration, and motor immaturity. FIRRST incorporates five elements of effective very early intervention described by Wallace and Rogers (19): 1) parent involvement, 2) frequency and length of intervention, 3) individualized and developmentally appropriate activities, 4) early initiation, and 5) focus on increasing parental sensitivity and responsiveness. Licensed health professionals trained by the developer delivered weekly 1-h sessions tailored to family routines and cultural practices. Each session comprises sharing progress, parent-child play with therapist's guidance and reflection and discussion of generalization and parent concerns. Sessions are videotaped for clinical supervision.

### 2.4 Outcome measures

#### 2.4.1 Primary outcome measure

##### 2.4.1.1 The measure of NDBI strategy implementation-caregiver change (MONSI-CC)

The MONSI-CC is an observational measure designed to evaluate caregivers' implementation of Naturalistic Developmental Behavioral Intervention (NDBI) strategies (18). The tool comprises 20 items organized into five domains—Environmental Set-Up, Child-Guided Interactions, Active Teaching and Learning, Opportunities for Social Communication, and Natural Reinforcement and Scaffolding—along with a composite score. In this study, the measure was applied to 10-min caregiver–child play sessions conducted in a clinical environment using a standardized set of toys. Each recording was viewed twice: initially to determine the child's play skills and language level, and subsequently for coding, during which the session was divided into two equal parts that were rated independently and then averaged to provide an estimate of caregiver strategy use. Coders were blind to assessment time point when scoring the video sessions. Items are scored on a 5-point scale (1 = lowest, 5 = highest) according to frequency and consistency, effectiveness of strategy use, and missed opportunities, with higher scores indicating more proficient and appropriate implementation.

#### 2.4.2 Secondary outcome measures

##### 2.4.2.1 Behavior observation of social communication change (BOSCC)

The BOSCC is an observational measure developed to capture changes in core autism symptoms in response to intervention (20). Derived from procedures used in the ADOS-2 (21), it comprises 12 items scored on a 6-point scale (0–5) and produces a Total score as well as Social Communication (SC) and Restricted and Repetitive Behaviors (RRB) subscale scores, with higher values indicating greater symptom atypicality. For the present study, BOSCC ratings were obtained from caregiver–child play interactions lasting 15 min and conducted with a standardized toy set. The middle 10 min of each interaction were coded, divided into two 5-min intervals that were scored independently and then averaged. Coders were blind to assessment time point when scoring the video sessions. The BOSCC shows solid inter-rater reliability, test–retest stability, and sensitivity to developmental and treatment-related change (20).

##### 2.4.2.2 Autism diagnostic observation schedule, second edition (ADOS-2)

The ADOS-2 is a standardized, semi-structured observational assessment used to evaluate social communication behaviors and restricted/repetitive behaviors associated with autism spectrum disorder (21, 22). The ADOS-2 includes five developmentally sequenced modules, selected based on the child's expressive language level and age, and provides structured opportunities for social interaction through play-based and conversational tasks. Each item is rated on a scale reflecting the degree of atypicality, and algorithm scores are derived to inform diagnostic classification. The ADOS-2 is widely regarded as a gold-standard measure for the behavioral assessment of ASD and shows strong reliability and validity across clinical and research settings. Consistent with ADOS-2 guidelines, the Toddler Module was administered only to children who met the relevant age and developmental eligibility criteria; accordingly, this measure was planned for a subset of participants and was not collected for the full sample.

##### 2.4.2.3 Griffiths scales of child development—third edition (griffiths III)

The Griffiths III is a standardized developmental assessment for children from birth to 6 years of age, widely used in clinical and research settings to evaluate early developmental functioning (23, 24). The tool assesses five domains: Foundations of Learning, Language and Communication, Eye–Hand Coordination, Personal–Social–Emotional, and Gross Motor. Each subscale yields raw, age-equivalent, and standardized scores (M = 100, SD = 15), and a General Developmental Score provides an overall estimate of developmental level. The Griffiths III has demonstrated strong reliability and validity and is frequently used in studies involving children with neurodevelopmental conditions, including ASD (25).

##### 2.4.2.4 Vineland adaptive behavior scales, second edition (VABS-II)

The VABS-II is a standardized caregiver-report measure used to assess adaptive functioning in everyday contexts (26). The instrument evaluates four core domains of adaptive behavior—Communication, Daily Living Skills, Socialization, and Motor Skills. The VABS-II is administered as a semi-structured interview and provides age-equivalent scores, standard scores, and adaptive composite scores based on normative data. The VABS-II has well-established reliability and validity and is widely used in clinical and research settings involving children with developmental disabilities (26–28).

##### 2.4.2.5 Parenting stress Index-4th edition (PSI-4)

The PSI-4 is a widely used screening and diagnostic questionnaire assessing stress experienced by parents of children with disabilities (29, 30). It consists of 85 items covering two domains: a Child Domain, which measures stress related to child characteristics (Mood, Acceptability/Distractibility, Adaptability/Demandingness, Reinforces Parent), and a Parent Domain, which measures stress related to parent characteristics (Spouse Support/Isolation, Incompetence/Guilt, Low Responsiveness, Life Restrictions). An optional Life Stress scale (19 items) provides information on stressors outside the parent–child relationship, such as separation, financial strain, or work-related problems. Scores between the 16th and 84th percentiles fall within the normal range; scores from the 85th to 89th percentile indicate elevated stress; and scores at or above the 90th percentile are considered clinically significant.

### 2.5 Data analysis

The distribution of the study variables met the assumptions of normality; thus, parametric analyses were conducted. A repeated measures analysis of variance (ANOVA) was used to assess changes in caregivers' implementation of NDBI strategies (MONSI-CC), autism symptomatology (BOSCC), developmental functioning (Griffiths III), adaptive behavior (Vineland-II), and parental stress (PSI-4) across three time points: baseline (T0), 3 months (T1), and 6 months post-intervention (T2). Statistical significance was set at *p* < .05.

## 3 Results

### 3.1 Feasibility and acceptability

**Figure 2:**
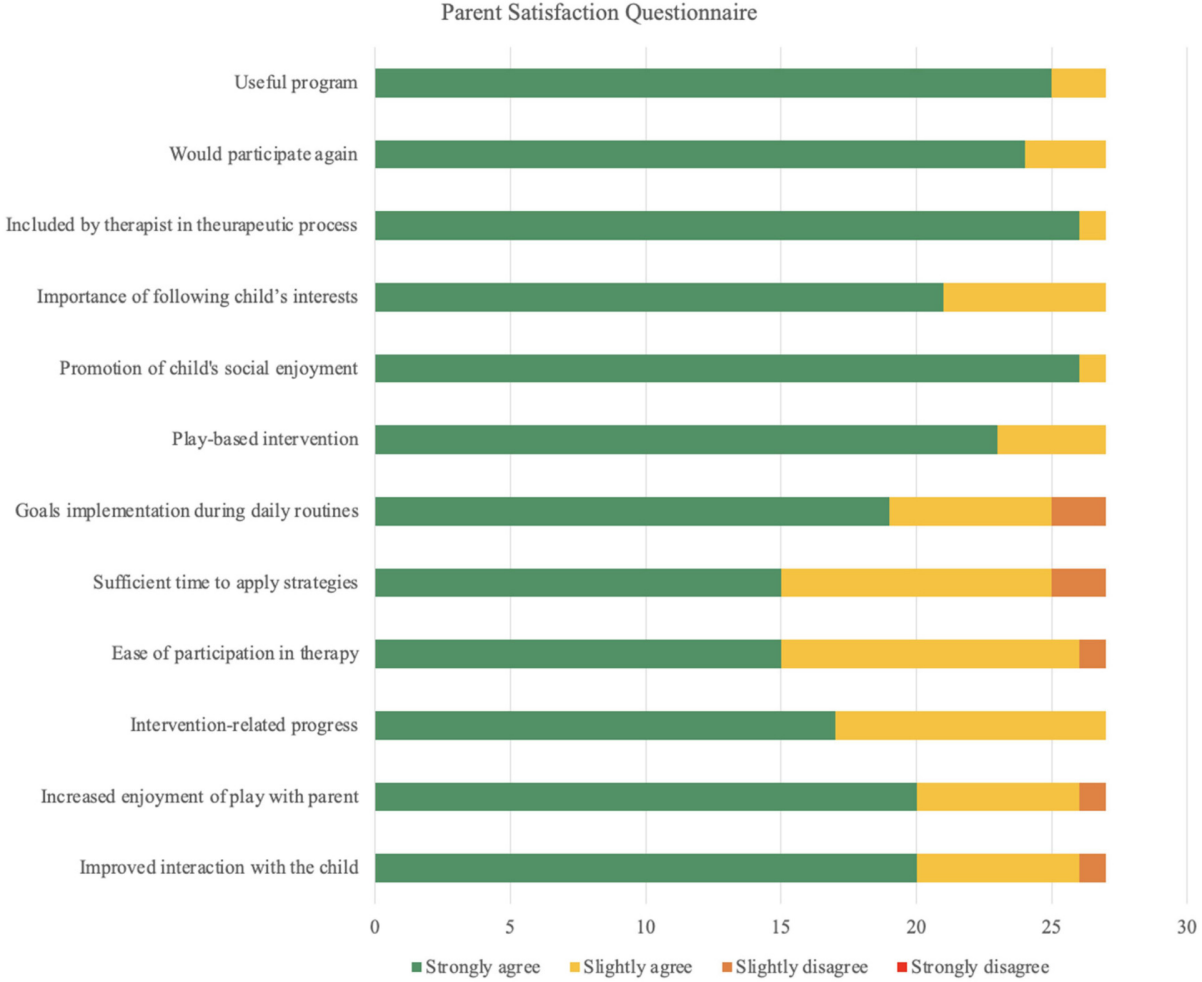
Parent satisfaction questionnaire.

Feasibility was supported by a very low attrition rate, with only 2 out of 30 families (6.7%) discontinuing participation. One family withdrew midway through the program due to the mother's return to work after maternity leave, which required frequent travel and prolonged absences from home, making continued involvement impractical. The second family completed all intervention sessions but did not attend the post-intervention evaluation (T2). No other families missed sessions or reported difficulties that interfered with participation. Taken together, these data indicate that the intervention was broadly feasible for the majority of families, with dropouts attributable to external practical constraints rather than issues related to the program itself. To evaluate acceptability, parents completed a satisfaction questionnaire at post-intervention; 27 out of 28 families returned the *measure*. Overall, parents reported high satisfaction with the intervention program. Across all items of the Satisfaction Questionnaire, the majority of respondents selected “*Strongly agree”* or “*Slightly agree”*, and no parents expressed strong disagreement with any statement. The highest levels of agreement were observed for: Promotion of the child's social enjoyment, Inclusion by the therapist in the therapeutic process, Perception of the program as useful, and Willingness to participate again. Slightly lower, though still positive, agreement levels were reported for items related to Ease of participation, Time to apply strategies, and Integration of therapy goals in routines, where a few parents chose “*Slightly agree”* or “*Slightly disagree”*. Overall, the pattern of responses indicates consistently positive perceptions of the intervention, suggesting that parents found the program useful and effective in promoting their child's social and interactive development (see Figure 2 for an overview of parental responses).

### 3.2 Pre-post intervention changes

#### 3.2.1 Primary outcome

##### 3.2.1.1 Caregiver implementation of NDBI strategies

**Figure 3:**
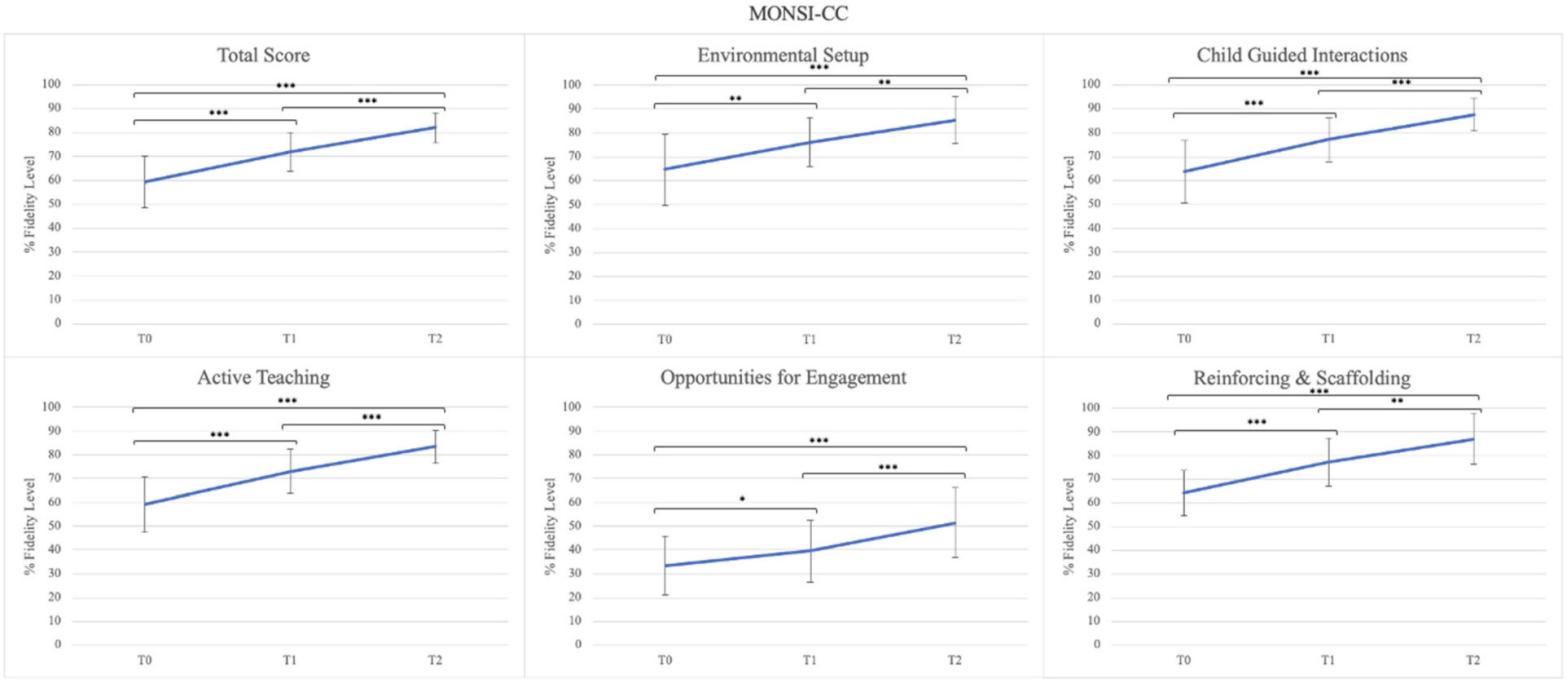
This figure illustrates the level of fidelity reached in the NDBI strategies measured by the MONSI-CC. Instead of using raw scores, percentages were calculated considering the total score achievable for each NDBI strategy of the MONSI-CC scale.*p < .05; **p < .01; ***p < .001.

For the MONSI-CC, significant main effects of Time were found for *Environmental Setup* [*F*(2,df) = 25.74, *p* < .001], *Child-Guided Interactions* [*F*(2,df) = 62.33, *p* < .001], *Active Teaching* [*F*(2,df) = 64.35, *p* < .001], *Opportunities for Engagement* [*F*(2, df) = 23.80, *p* < .001], *Reinforcing and Scaffolding* [*F*(2, df) = 51.68, *p* < .001], and the *Total Score* [*F*(2, df) = 67.98, *p* < .001]. Bonferroni *post hoc* comparisons showed progressive increases across all domains from T0 to T2, with all pairwise differences reaching significance (all *p* < .05) (an overview of results is presented in Table 2 and Figure 3).

**Table 2:** Differences over time in child and parental measures using repeated measures ANOVA.

Outcome measures										Bonferroni *post-hoc* comparisons								
	T0		T1		T2		ANOVA			T0-T1			T0-T2			T1-T2		
	M	(SD)	M	(SD)	M	(SD)	*F*	*p* value	*η_p_* ^2^	MD (SE)	*p* value	Cohen's *d*	MD (SE)	*p* value	Cohen's *d*	MD (SE)	*p* value	Cohen's *d*
MONSI-CC																		
Environmental setup	9.71	(2.24)	11.43	(1.54)	12.79	(1.48)	25.74	<.001***	0.49	−1.71 (.40)	.001**	1.35	−3.07 (.48)	<.001***	2.11	−1.36 (.40)	.006**	1.01
Child guided interactions	15.93	(3.23)	19.29	(2.32)	21.89	(1.69)	62.33	<.001***	0.70	−3.58 (.50)	<.001***	1.29	−5.96 (.56)	<.001***	2.30	−2.61 (.54)	<.001***	1.38
Active teaching	20.73	(4.07)	25.55	(3.26)	29.25	(2.43)	64.35	<.001***	0.70	−4.82 (.75)	<.001***	1.44	−8.52 (.80)	<.001***	3.02	−3.70 (.70)	<.001***	1.40
Opportunities for engagement	3.34	(1.23)	3.95	(1.31)	5.14	(1.46)	23.80	<.001***	0.47	−.61 (.21)	.023*	0.54	−1.80 (.31)	<.001***	1.47	−1.20 (.27)	<.001***	1.36
Reinforcing & scaffolding	9.63	(1.42)	11.59	(1.53)	13.05	(1.58)	51.68	<.001***	0.66	−1.96 (.27)	<.001***	1.48	−3.43 (.36)	<.001***	2.53	−1.46 (.38)	.002**	1.05
Total score	59.34	(10.70)	71.80	(8.12)	82.13	(6.26)	67.98	<.001***	0.72	−12.46 (1.84)	<.001***	1.43	−22.79 (2.15)	<.001***	3.06	−1.32 (1.87)	<.001***	1.56
BOSCC																		
Social-communication	29.14	(5.07)	18.30	(5.56)	12.71	(6.99)	76.58	<.001***	0.74	1.84 (1.17)	<.001***	−2.27	16.43 (1.32)	<.001***	−2.90	5.59 (1.53)	.003**	−0.97
Restricted and repetitive behaviors	5.00	(1.87)	3.57	(1.04)	2.77	(1.27)	24.03	<.001***	0.47	1.43 (.34)	.001**	−0.95	2.32 (.36)	<.001***	−1.48	.80 (.27)	.019*	−0.76
Total score	34.14	(5.24)	22.82	(6.04)	15.48	(7.79)	82.19	<.001***	0.75	11.32 (1.22)	<.001***	−2.22	7.34 (1.64)	<.001***	−2.98	18.66 (1.51)	<.001***	−1.15
Griffiths III																		
Foundations of learning	95.04	(13.52)	100.96	(14.37)	104.68	(10.64)	5.08	.010*	0.18	−5.92 (2.75)	.126	0.47	−9.64 (2.30)	.011*	1.27	−3.72 (3.37)	.842	0.32
Language and communication	83.84	(12.03)	96.76	(9.61)	103.28	(8.80)	39.52	<.001***	0.62	−12.92 (2.52)	<.001***	1.30	−19.44 (2.49)	<.001***	2.39	−6.52 (1.53)	.001**	1.19
Eye and hand coordination	92.92	(11.03)	101.32	(11.90)	105.92	(11.70)	9.02	<.001***	0.27	−8.4 (3.23)	.047	1.22	−13,00 (3.12)	.001**	1.28	−4.6 (2.95)	.397	0.44
Personal-social-emotional	82.88	(10.94)	94.88	(10.01)	103.04	(7.68)	31.75	<.001***	0.57	−12,00 (2.48)	<.001***	1.28	−2.16 (2.95)	<.001***	2.28	−8.16 (2.14)	.003**	1.00
Gross motor	97.56	(9.77)	97.80	(13.28)	102.56	(16.51)	1.70	.193	0.07	−.24 (2.58)	1.000	0.02	−5.00 (3.17)	.385	0.38	−4.76 (3.36)	.508	0.35
General development	88.60	(9.62)	96.92	(9.63)	103.72	(9.59)	27.99	<.001***	0.54	−8.32 (1.84)	<.001***	1.37	−15.12 (2.31)	<.001***	2.16	−6.80 (1.88)	.004**	1.19
Vineland-II																		
Communication	84.69	(7.55)	92.00	(8.76)	99.27	(8.76)	28.67	<.001***	0.53	−7.31 (1.80)	.001**	1.39	−14.58 (1.97)	<.001***	2.38	−7.27 (2.00)	.004**	1.33
Daily living skills	87.96	(11.19)	96.00	(8.61)	102.19	(10.93)	20.74	<.001***	0.45	−8.04 (2.29)	.005**	1.28	−14.23 (2.48)	<.001***	1.44	−6.19 (1.84)	.007**	1.09
Socialization	88.00	(10.64)	90.69	(8.84)	98.50	(8.76)	18.40	<.001***	0.42	−2.69 (1.97)	.548	0.30	−1.50 (1.85)	<.001***	1.19	−7.81 (1.56)	<.001***	1.39
Motor skills	91.04	(13.17)	92.96	(10.46)	100.77	(11.46)	6.64	.003*	0.21	−1.92 (3.27)	1.000	0.18	−9.73 (2.38)	.001**	1.27	−7.81 (2.77)	.028*	1.19
Adaptive behavior composite	88.15	(10.38)	91.19	(8.33)	100.04	(10.18)	14.28	<.001***	0.36	−3.04 (2.67)	.798	0.35	−11.89 (2.18)	<.001***	1.29	−8.85 (2.03)	.001**	1.05
PSI-4																		
Total stress	77.21	(24.88)	75.47	(30.90)	77.89	(29.16)	0.26	.712	0.01	1.74 (4.12)	1.000	−0.07	−.68 (3.84)	1.000	0.03	2.42 (2.28)	.906	0.09
Parent domain	76.84	(26.37)	74.11	(30.75)	79.05	(25.88)	0.93	.403	0.05	2.74 (3.73)	1.000	−0.11	−2.21 (4.31)	1.000	0.09	−4.95 (2.66)	.237	0.19
Spouse support/isolation	77.11	(25.25)	78.74	(26.61)	81.16	(24.33)	0.81	.452	0.04	−1.63 (3.27)	1.000	0.07	−4.05 (3.70)	.865	0.18	−2.42 (2.52)	1.000	0.11
Incompetence/guilt	74.05	(25.20)	69.95	(26.58)	70.84	(26.53)	0.63	.540	0.03	4.11 (4.29)	1.000	−0.18	3.21 (4.33)	1.000	−0.14	−.90 (2.72)	1.000	0.04
Low responsiveness	71.21	(28.13)	74.42	(27.60)	71.42	(25.59)	0.24	.789	0.01	−3.21 (3.73)	1.000	0.13	−.21 (5.70)	1.000	0.01	3.00 (5.82)	1.000	−0.13
Life restrictions	74.89	(29.92)	69.89	(35.26)	77.16	(28.98)	1.29	.287	0.07	5.00 (4.81)	.937	−0.17	−2.26 (4.96)	1.000	0.09	7.26 (4.05)	.269	0.25
Child domain	76.37	(28.26)	74.21	(29.41)	74.79	(30.76)	0.20	.820	0.01	2.16 (4.38)	1.000	−0.08	1.58 (3.41)	1.000	−0.06	−.58 (2.97)	1.000	0.02
Mood	68.74	(26.86)	65.79	(30.78)	75.32	(26.14)	4.02	.040*	0.18	2.95 (3.22)	1.000	−0.11	6.58 (2.60)	.063	0.28	9.53 (4.28)	.117	0.37
Acceptability/distraibility	81.11	(23.53)	79.68	(24.62)	73.95	(28.96)	2.79	.075	0.13	1.42 (2.92)	1.000	−0.07	7.16 (2.75)	.054	−0.30	5.74 (3.86)	.463	−0.24
Adaptability/demandness	73.58	(28.88)	73.63	(27.99)	74.37	(29.58)	0.02	.942	0.001	−.05 (4.47)	1.000	0.00	−.79 (5.42)	1.000	0.03	−.74 (2.72)	1.000	0.03
Reinforces parent	68.95	(33.39)	63.42	(31.04)	62.16	(30.51)	0.78	.468	0.04	5.53 (6.51)	1.000	−0.19	6.79 (6.22)	.869	−0.24	1.26 (4.46)	1.000	−0.05

* *p* < .05.

** *p* < .01.

*** *p* < .001.

*P*-value adjusted for comparing a family of 3. Effect size magnitude should be interpreted using conventional benchmarks (small = 0.20, medium = 0.50, large = 0.80). Values ≥1.50 should be considered very large, indicating effects of substantial magnitude.

.

#### 3.2.2 Secondary outcomes

##### 3.2.2.1 Changes in children's ASD core symptom

**Figure 4:**
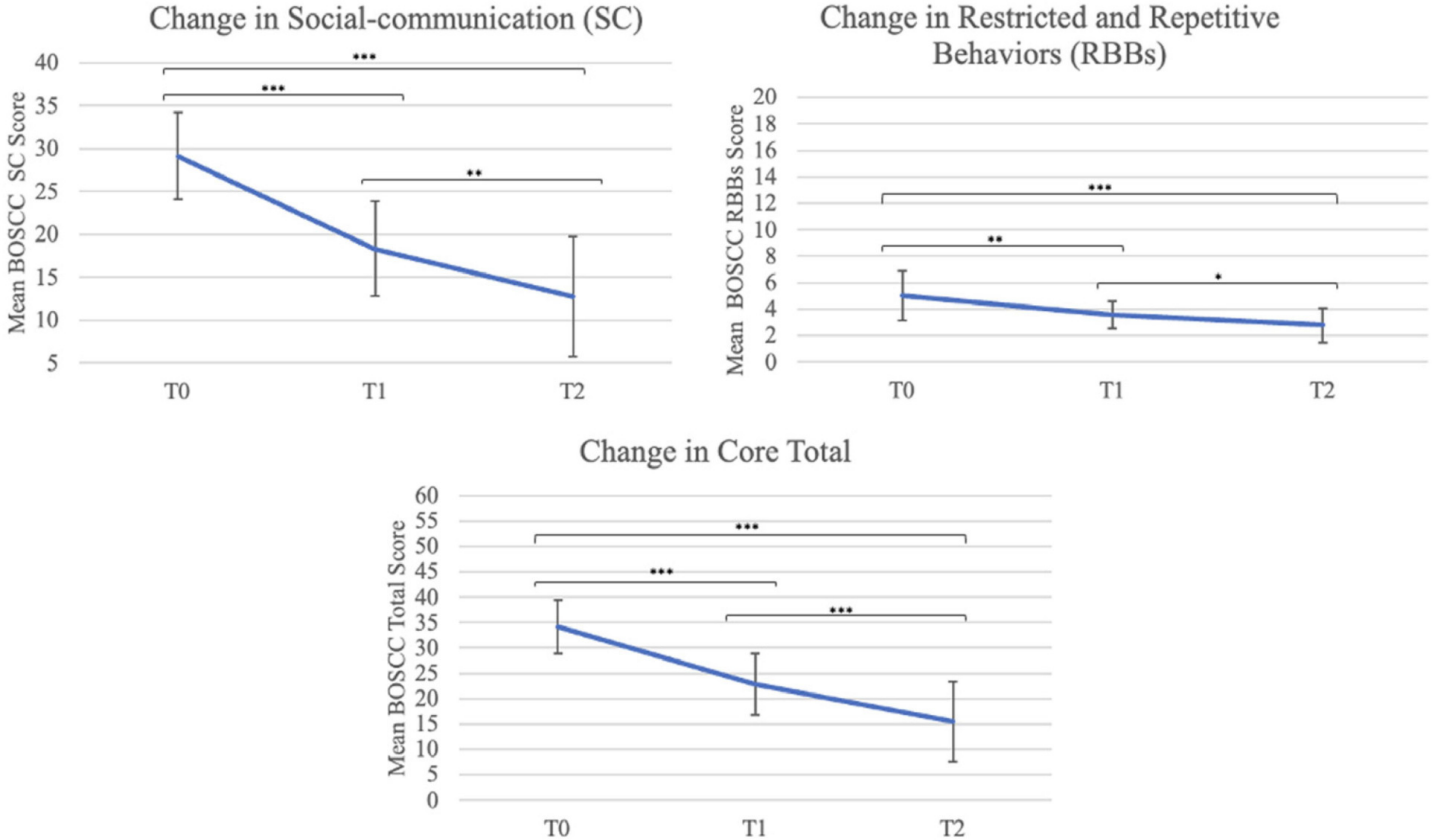
Changes in BOSCC domains over time. *p < .05; **p < .01; ***p < .001.

For the BOSCC, significant effects of Time emerged for *Social Communication* [*F*(2,df) = 76.58, *p* < .001], *Restricted and Repetitive Behaviors* [F(2,df) = 24.03, *p* < .001], and *Total Score* [*F*(2,df) = 82.19, *p* < .001]. *post hoc* comparisons indicated significant reductions between all time points (all *p* < .05) (see Figure 4).

##### 3.2.2.2 Changes in children's development

**Figure 5:**
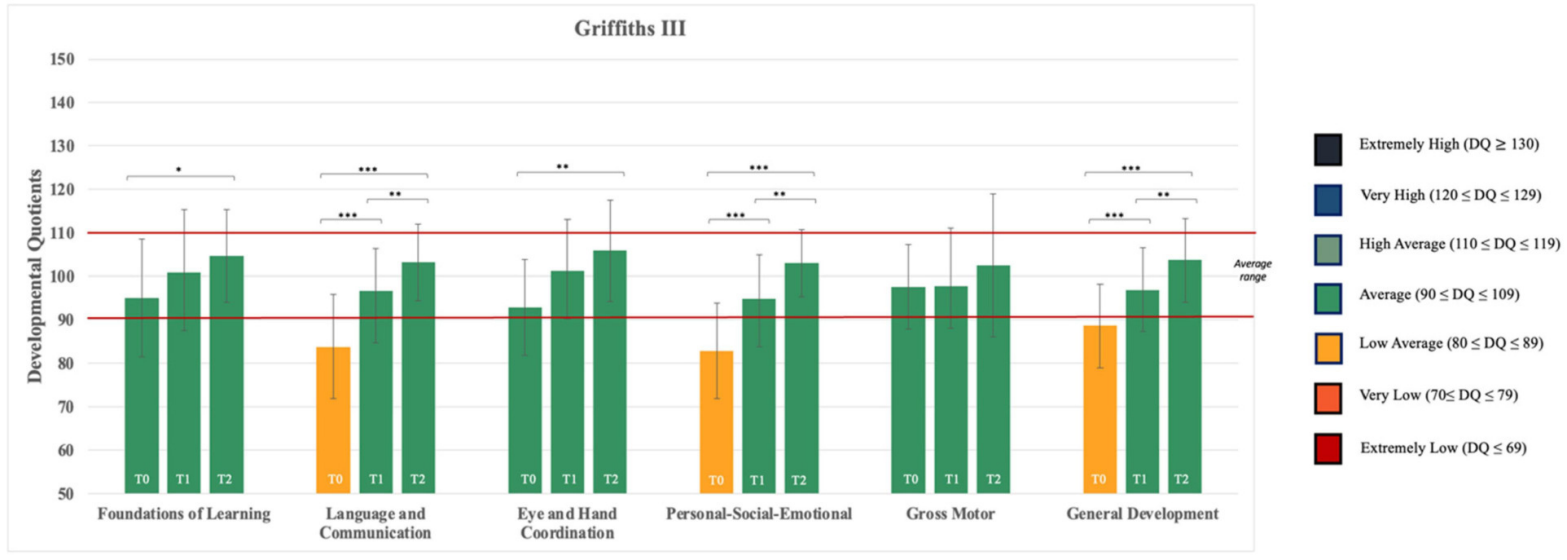
Changes in griffiths III subscales over time. *p < .05; **p < .01; ***p < .001.

For the Griffiths III, significant main effects of Time were found for *Foundations of Learning* [*F*(2,df) = 5.08, *p* = .010], *Language and Communication* [*F*(2,df) = 39.52, *p* < .001], *Eye and Hand Coordination* [*F*(2,df) = 9.02, *p* < .001], *Personal–Social–Emotional* [*F*(2,df) = 31.75, *p* < .001], and *General Development* [*F*(2,df) = 27.99, *p* < .001], while *Gross Motor* was not significant [*F*(2,df) = 1.70, *p* = .193]. Bonferroni tests revealed significant increases from T0 to T2 for all significant domains (all *p* < .05) (see Figure 5).

##### 3.2.2.3 Changes in children's adaptive behavior

**Figure 6:**
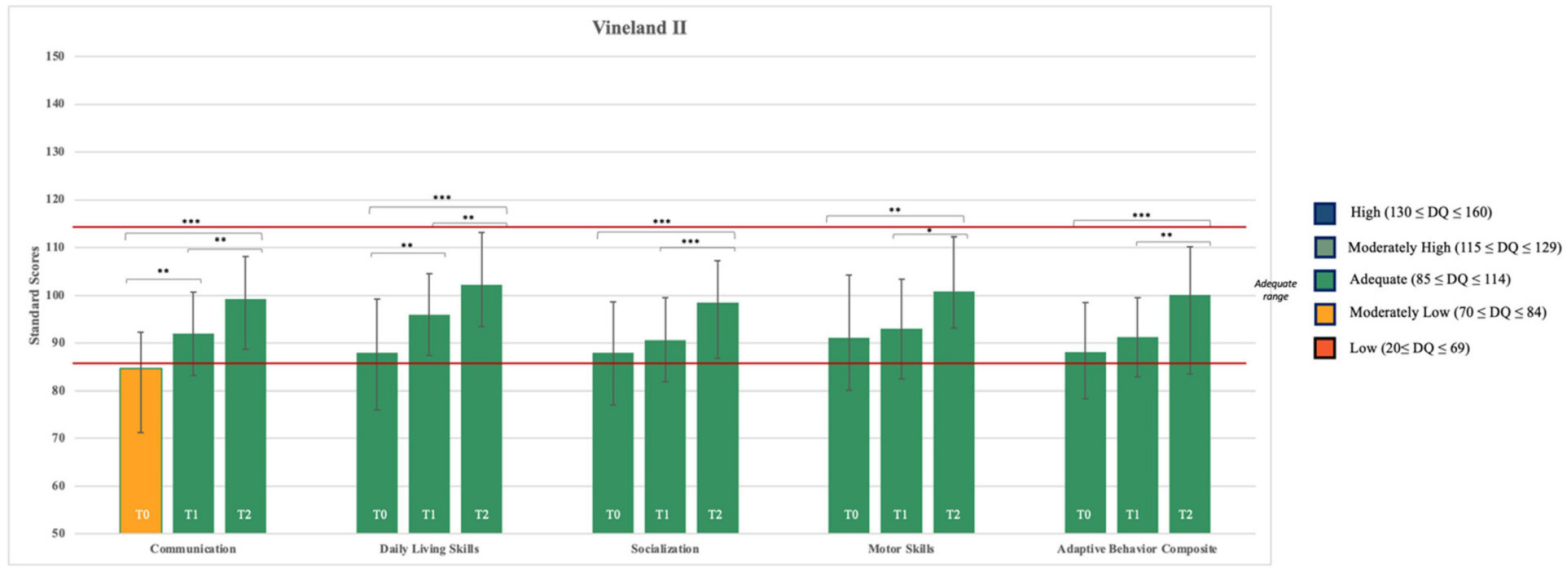
Changes in vineland-II domains over time. *p < .05; **p < .01; ***p < .001.

For the Vineland-II, significant main effects of Time were observed for *Communication* [*F*(2,df) = .67, *p* < .001], *Daily Living Skills* [*F*(2,df) = .27.74, *p* < .001], *Socialization* [*F*(2,df) = 18.40, *p* < .001], *Motor Skills* [*F*(2,df) = 6.64, *p* = .003], and *Adaptive Behavior Composite* [*F*(2,df) = .14.28, *p* < .001]. *Post hoc* analyses indicated significant improvements between T0 and T2 for all domains (all *p* < .01) (see Figure 6).

##### 3.2.2.4 Changes in parents' stress levels

For the PSI-4 no significant main effects of Time were found for the *Total Stress* score [*F*(2,df) *=* *.*26, *p* = .712] or for the subscales (*p* > .05), except for *Mood* [*F*(2,df) *=* 4.02, *p* = .040], where *post hoc* comparisons did not reach corrected significance.

##### 3.2.2.5 Autism symptoms at post-intervention assessment

**Figure 7:**
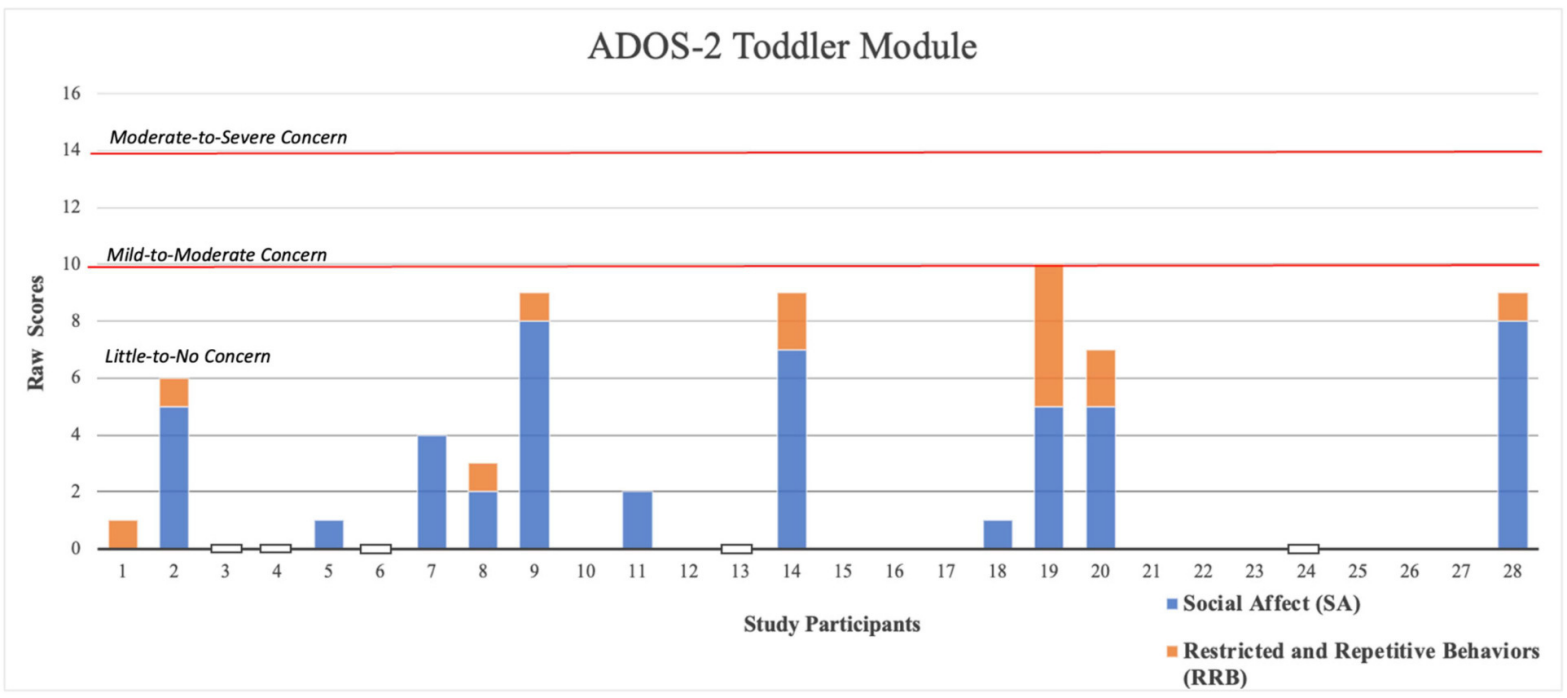
Overview of ados-2 toddler module scores for each participant.

At Post Intervention (T2), 17 out of 28 children were able to undergo the ADOS-2 Toddler Module (they were >12 months and able to walk). ADOS-2 Toddler Module scores are reported for the subset of children who met the eligibility criteria for this module, reflecting planned assessment constraints rather than missing data. The mean ADOS Total score was 3.35 (SD = 3.97), the Social Affect (SA) mean was 2.82 (SD = 2.98), and the Restricted and Repetitive Behaviors (RRB) mean was 0.82 (SD = 1.29) (see Figure 7).

## 4 Discussion

The present pilot study provides initial support for the feasibility and potential benefits of FIRRST, a telehealth, parent-mediated NDBI program for infants under 15 months with early signs of ASD, implemented within routine Italian clinical services. Families showed high retention and satisfaction, and caregivers reached progressively higher fidelity in NDBI strategies, accompanied by improvements in children's social-communication, developmental functioning, and adaptive behavior and a decrease in ASD symptoms. These findings extend previous work on preemptive interventions such as Infant Start and iBASIS-VIPP (12, 13) by demonstrating that a first-year-of-life, parent-mediated model can be delivered in a real-world tertiary service, outside of a tightly controlled research environment (9, 17).

Beyond demonstrating feasibility and preliminary clinical impact, these findings highlight the potential value of systematically intercepting early ASD symptoms from the first year of life, when subtle but reliable social-communication atypicalities become observable. Identifying infants with prodromal signs in this developmental window and establishing clear referral pathways from pediatric care to specialized services can support a detect and treat prodromes approach, in which intervention is initiated on the basis of early vulnerability rather than waiting for a full diagnostic profile to consolidate. Embedding preemptive, parent-mediated programs like FIRRST within routine services may therefore help reduce delays between the emergence of early signs and access to targeted support, with possible downstream benefits for developmental and adaptive trajectories.

Consistent with prior studies on parent-mediated NDBIs and Parent-ESDM, caregivers in FIRRST substantially increased their use of responsive, child-guided and active teaching strategies over the six-month intervention (14, 17, 18). The magnitude and profile of change in MONSI-CC scores are comparable to those reported in older toddlers receiving parent-mediated interventions, suggesting that parents of symptomatic infants can rapidly acquire complex interactional skills when supported through individualized coaching (18, 31). Parallel reductions in BOSCC-rated ASD symptoms and gains on the Griffiths III and Vineland-II align with the broader literature indicating that parent-mediated NDBIs can positively influence core social-communication behaviors and developmental milestones, even at relatively low intensity (20, 32, 33). The absence of significant change in parental stress echoes mixed findings from previous parent-mediated trials and may reflect the dual impact of increased demands and increased competence among caregivers of very young, ASD high likelihood infants (34, 35).

### 4.1 Strengths

A key strength of this study is that FIRRST was implemented within a public clinical service, with procedures and resources closely aligned to usual care in an Italian tertiary hospital. This real-world implementation increases ecological validity and complements the evidence derived from highly controlled RCTs conducted in research-intensive settings, where staffing levels, training, and supervision may not be easily replicated in routine practice (12, 13, 36). In line with recent community-based work on ESDM and Social ABCs, the present results suggest that parent-mediated, evidence-based models can be integrated into existing service pathways and can reach families at the time when neural plasticity and developmental sensitivity are highest (15, 16, 37, 38).

A second strength lies in the systematic use of validated observational and standardized measures across multiple domains, including MONSI-CC, BOSCC, Griffiths III, Vineland-II, and ADOS-2 Toddler Module. This multi-informant, multi-method approach provides a coherent picture of change in both caregiver behavior and child outcomes, and follows recommendations for outcome selection in early ASD intervention research (20, 39). Furthermore, the telehealth format proved feasible for families of infants under 15 months, supporting prior evidence that remote coaching can be an effective and scalable modality for delivering parent-mediated interventions (40, 41). This may be especially relevant for families encountering logistical or environmental constraints that make weekly visits to specialized centers difficult, such as those residing in rural contexts.

### 4.2 Limitations and future directions

Several limitations must be acknowledged. First, the single-arm design and modest sample size prevent causal inferences about efficacy and limit generalizability. As in other feasibility studies of parent-mediated interventions (17, 42), improvements could partly reflect maturation, regression to the mean, or concurrent community services, despite the low likelihood of intensive specialized intervention at this age within the Italian system (43, 44). Second, while the sample is clinically representative of infants referred to a tertiary service and selected via SACS-R ASD likelihood scores, it does not include families from more diverse socio-economic or cultural backgrounds and may underestimate implementation challenges in lower-resourced contexts (11, 42). Third, the current report focuses on short-term outcomes over six months; longer follow-up is needed to determine whether early gains in social-communication and development translate into sustained reductions in ASD symptom severity, diagnostic outcomes, and service use (5, 45).

To address these limitations, a multicenter randomized controlled trial of FIRRST vs. a structured Parent Education condition is currently underway, with a larger sample, blinded assessments, and *a priori* power to detect clinically meaningful between-group differences in ASD symptoms, developmental quotients, caregiver-child interaction, and neurophysiological markers (ClinicalTrials.gov NCT06817746). This RCT will build directly on the feasibility data presented here, testing the efficacy and mechanisms of FIRRST in comparison with an active control, in line with international guidelines and recent RCTs of preemptive interventions in infants at high likelihood for ASD (12, 13, 36). In parallel, longitudinal follow-up of the pilot cohort is planned to characterize developmental, adaptive, and diagnostic trajectories into the preschool years, and to examine whether early changes in NDBI strategy use and infant social-communication predict later outcomes, as suggested by prior work on infant siblings and infants at high likelihood for ASD (4, 7, 45).

### 4.3 Conclusions

Within the broader shift from a “diagnose then treat” model to a “detect and treat prodromes” approach in ASD care, FIRRST represents an important translational step toward embedding preemptive, parent-mediated intervention for symptomatic infants into routine clinical services. The present findings indicate that such a telehealth NDBI program is feasible, acceptable to families, and associated with clinically meaningful improvements in caregiver interaction strategies and early child outcomes, thereby providing a strong rationale for rigorous evaluation in an ongoing randomized controlled trial (9, 12, 13, 17). If supported by forthcoming RCT and longitudinal data, FIRRST could contribute to real-world implementation of very early, scalable interventions that reduce delays in access to care and potentially mitigate long-term disability and service burden associated with ASD.

## Data Availability

The raw data supporting the conclusions of this article will be made available by the authors, without undue reservation.

## References

[1] American Psychological Association. Diagnostic and Statistical Manual of Mental Disorders. 5th ed., text rev. Washington (2022). 10.1176/appi.books.9780890425787

[2] Ostrowski J Religioni U Gellert B Sytnik-Czetwertyński J Pinkas J. Autism Spectrum disorders: etiology, epidemiology, and challenges for public health. Med Sci Monit. (2024) 30:e944161. 10.12659/MSM.944161 38833427 PMC11162141

[3] Hiremath CS Sagar KJV Yamini BK Girimaji AS Kumar R Sravanti SL. Emerging behavioral and neuroimaging biomarkers for early and accurate characterization of autism spectrum disorders: a systematic review. Transl Psychiatry. (2021) 11(1):42. 10.1038/s41398-020-01178-6 33441539 PMC7806884

[4] Cleary DB, Maybery MT, Green C, Whitehouse AJO. The first six months of life: a systematic review of early markers associated with later autism. Neurosci Biobehav Rev. (2023) 152:105304. 10.1016/j.neubiorev.2023.105304 37406749

[5] Lombardo MV Busuoli EM Schreibman L Stahmer AC Pramparo T Landi I. Pre-treatment clinical and gene expression patterns predict developmental change in early intervention in autism. Mol Psychiatry. (2021) 26(12):7641–51. 10.1038/s41380-021-01239-2 34341515 PMC8872998

[6] Guthrie W Wetherby AM Woods J Schatschneider C Holland RD Morgan L. The earlier the better: an RCT of treatment timing effects for toddlers on the autism spectrum. Autism. (2023) 27(8):13623613231159153. 10.1177/13623613231159153 36922406 PMC10502186

[7] Jones W Klin A. Attention to eyes is present but in decline in 2–6-month-old infants later diagnosed with autism. Nature. (2013) 504(7480):427–31. 10.1038/nature12715 24196715 PMC4035120

[8] Bosl WJ Tager-Flusberg H Nelson CA. EEG analytics for early detection of autism Spectrum disorder: a data-driven approach. Sci Rep. (2018) 8(1):6828. 10.1038/s41598-018-24318-x 29717196 PMC5931530

[9] Colombi C Chericoni N Bargagna S Costanzo V Devescovi R Lecciso F. Case report: preemptive intervention for an infant with early signs of autism spectrum disorder during the first year of life. Front Psychiatry. (2023) 14:1105253. 10.3389/fpsyt.2023.1105253 37205979 PMC10189150

[10] Barbaro J Dissanayake C. Early markers of autism spectrum disorders in infants and toddlers prospectively identified in the social attention and communication study. Autism. (2013) 17(1):64–86. 10.1177/1362361312442597 22735682

[11] Barbaro J Sadka N Gilbert M Beattie E Li X Ridgway L. Diagnostic accuracy of the social attention and communication surveillance-revised with preschool tool for early autism detection in very young children. JAMA Netw Open. (2022) 5(3):e2146415. 10.1001/jamanetworkopen.2021.46415 35275169 PMC8917423

[12] Rogers SJ Vismara L Wagner A McCormick C Young G Ozonoff S. Autism treatment in the first year of life: a pilot study of infant start, a parent-implemented intervention for symptomatic infants. J Autism Dev Disord. (2014) 44(12):2981–95. 10.1007/s10803-014-2202-y 25212413 PMC4951093

[13] Whitehouse AJO Varcin KJ Pillar S Billingham W Alvares GA Barbaro J. Effect of preemptive intervention on developmental outcomes among infants showing early signs of autism: a randomized clinical trial of outcomes to diagnosis. JAMA Pediatr. (2021) 175(11):e213298. 10.1001/jamapediatrics.2021.3298 34542577 PMC8453361

[14] Schreibman L Dawson G Stahmer AC Landa R Rogers SJ McGee GG. Naturalistic developmental behavioral interventions: empirically validated treatments for autism Spectrum disorder. J Autism Dev Disord. (2015) 45(8):2411–28. 10.1007/s10803-015-2407-8 25737021 PMC4513196

[15] Colombi C Narzisi A Ruta L Cigala V Gagliano A Pioggia G. Implementation of the early start denver model in an Italian community. Autism. (2018) 22(2):126–33. 10.1177/1362361316665792 29110508

[16] Devescovi R Colonna V Dissegna A Bresciani G Carrozzi M Colombi C. Feasibility and outcomes of the early start denver model delivered within the public health system of the friuli venezia giulia Italian region. Brain Sci. (2021) 11(9):1191. 10.3390/brainsci11091191 34573216 PMC8464931

[17] Chericoni N Colombino I Conti E Guainai G Riva B Qu L. Feasibility of an evidence-based parent-mediated intervention for autism Spectrum disorder in a community healthcare service in Italy. Children. (2025) 12(12):1651. 10.3390/children12121651 41462792 PMC12731391

[18] Vibert BA Dufek S Klein CB Choi YB Winter J Lord C. Quantifying caregiver change across early autism interventions using the measure of NDBI strategy implementation: caregiver change (MONSI-CC). J Autism Dev Disord. (2020) 50(4):1364–79. 10.1007/s10803-019-04342-0 31925669 PMC7103564

[19] Wallace KS Rogers SJ. Intervening in infancy: implications for autism spectrum disorders. J Child Psychol Psychiatry. (2010) 51(12):1300–20. 10.1111/j.1469-7610.2010.02308.x 20868374 PMC4928976

[20] Grzadzinski R Carr T Colombi C McGuire K Dufek S Pickles A. Measuring changes in social communication behaviors: preliminary development of the brief observation of social communication change (BOSCC). J Autism Dev Disord. (2016) 46(7):2464–79. 10.1007/s10803-016-2782-9 27062034

[21] Lord C Luyster RJ Gotham K Guthrie W. Autism Diagnostic Observation Schedule. 2nd ed. Torrance, CA: Western Psychological Services (2012).

[22] Colombi C Tancredi R Persico A Faggioli R. ADOS-2—autism Diagnostic Observation Schedule. 2nd ed. Firenze: Hogrefe (2013).

[23] Green E Stroud L Bloomfield S Cronje J Foxcroft C Hurter K. Griffith III-Griffths Scale of Child Developmental. Florence, Italy: Hogrefe (2016).

[24] Strisciuglio P Griffiths R. Griffiths III—scale griffiths dello sviluppo del bambino. In: Hogrefe F, editor. Manuale. Firenze: Hogrefe (2016).

[25] Lecciso F Martis C Levante A. The use of griffiths III in the appraisal of the developmental profile in autism: a systematic search and review. Brain Sci. (2025) 15(5):506. 10.3390/brainsci15050506 40426677 PMC12110223

[26] Sparrow SS. In: Cicchetti DV, editor. Vineland Adaptive Behavior Scales, Second Edition (Vineland-II). London: Pearson (2005).

[27] Balboni G Tasso A Muratori F Cubelli R. The vineland-II in preschool children with autism spectrum disorders: an item content category analysis. J Autism Dev Disord. (2016) 46(1):42–52. 10.1007/s10803-015-2533-3 26210516

[28] Yang S Paynter JM Gilmore L. Vineland adaptive behavior scales: iI profile of young children with autism spectrum disorder. J Autism Dev Disord. (2016) 46(1):64–73. 10.1007/s10803-015-2543-1 26231205

[29] Abidin RR. Parenting stress index–fourth edition (PSI-4). Lutz, FL Psychol Assess Resour. (2012) 3:1–16.

[30] Guarino A Laghi F Serantoni G Di Blasio P Camisasca E. Parenting Stress Index–Fourth Edition (PSI-4). Giunti O.S. Firenze (2016).

[31] Rogers SJ Estes A Lord C Vismara L Winter J Fitzpatrick A. Effects of a brief early start denver model (ESDM)-based parent intervention on toddlers at risk for autism spectrum disorders: a randomized controlled trial. J Am Acad Child Adolesc Psychiatry. (2012) 51(10):1052–65. 10.1016/j.jaac.2012.08.003 23021480 PMC3487718

[32] Dawson G Rogers S Munson J Smith M Winter J Greenson J. Randomized, controlled trial of an intervention for toddlers with autism: the early start denver model. Pediatrics. (2010) 125(1):e17–23. 10.1542/peds.2009-0958 19948568 PMC4951085

[33] Oono IP Honey EJ McConachie H. Parent-mediated early intervention for young children with autism spectrum disorders (ASD). Cochrane Database Syst Rev. (2013) 2013(4):CD009774. 10.1002/14651858.CD009774.pub2 23633377 PMC11831248

[34] Estes A Vismara L Mercado C Fitzpatrick A Elder L Greenson J. The impact of parent-delivered intervention on parents of very young children with autism. J Autism Dev Disord. (2014) 44(2):353–65. 10.1007/s10803-013-1874-z 23838727 PMC3888483

[35] Jurek L Leadbitter K Falissard B Colin C Touzet S Geoffray MM. Parental experience of parent-mediated intervention for children with ASD: a systematic review and qualitative evidence synthesis. Autism. (2023) 27(3):647–66. 10.1177/13623613221112204 35899918

[36] Green J Charman T Pickles A Wan MW Elsabbagh M Slonims V. Parent-mediated intervention versus no intervention for infants at high risk of autism: a parallel, single-blind, randomised trial. Lancet Psychiatry. (2015) 2(2):133–40. 10.1016/S2215-0366(14)00091-1 26359749 PMC4722333

[37] Brian J Drmic I Roncadin C Dowds E Shaver C Smith IM. Effectiveness of a parent-mediated intervention for toddlers with autism spectrum disorder: evidence from a large community implementation. Autism. (2022) 26(7):1882–97. 10.1177/13623613211068934 35037520

[38] Landa RJ. Efficacy of early interventions for infants and young children with, and at risk for, autism spectrum disorders. Int Rev Psychiatry. (2018) 30(1):25–39. 10.1080/09540261.2018.1432574 29537331 PMC6034700

[39] Sandbank M Bottema-Beutel K Woynaroski T. Intervention recommendations for children with autism in light of a changing evidence base. JAMA Pediatr. (2021) 175(4):341–2. 10.1001/jamapediatrics.2020.4730 33165523

[40] Vismara LA McCormick CEB Wagner AL Monlux K Nadhan A Young GS. Telehealth parent training in the early start denver model: results from a randomized controlled study. Focus Autism Other Dev Disabil. (2018) 33(2):67–79. 10.1177/1088357616651064

[41] Ingersoll B Frost KM Straiton D Ramos AP Casagrande K. Telehealth coaching in project ImPACT indirectly affects children’s expressive language ability through parent intervention strategy use and child intentional communication: an RCT. Autism Res. (2024) 17(10):2177–87. 10.1002/aur.3230 39233512

[42] Rogers SJ Stahmer A Talbott M Young G Fuller E Pellecchia M. Feasibility of delivering parent-implemented NDBI interventions in low-resource regions: a pilot randomized controlled study. J Neurodev Disord. (2022) 14(1):3. 10.1186/s11689-021-09410-0 34986782 PMC8903494

[43] Borgi M Ambrosio V Cordella D Chiarotti F Venerosi A. Nationwide survey of healthcare services for autism Spectrum disorders (ASD) in Italy. Adv Neurodev Disord. (2019) 3(3):306–18. 10.1007/s41252-019-00113-1

[44] Salomone E Beranová Š Bonnet-Brilhault F Briciet Lauritsen M Budisteanu M Buitelaar J. Use of early intervention for young children with autism spectrum disorder across Europe. Autism. (2016) 20(2):233–49. 10.1177/1362361315577218 25916866

[45] Green J Pickles A Pasco G Bedford R Wan MW Elsabbagh M. Randomised trial of a parent-mediated intervention for infants at high risk for autism: longitudinal outcomes to age 3 years. J Child Psychol Psychiatry. (2017) 58(12):1330–40. 10.1111/jcpp.12728 28393350 PMC5724485

